# Prey capture in zebrafish larvae serves as a model to study cognitive functions

**DOI:** 10.3389/fncir.2013.00110

**Published:** 2013-06-11

**Authors:** Akira Muto, Koichi Kawakami

**Affiliations:** ^1^Division of Molecular and Developmental Biology, National Institute of GeneticsMishima, Shizuoka, Japan; ^2^Department of Genetics, The Graduate University for Advanced Studies (SOKENDAI)Mishima, Shizuoka, Japan

**Keywords:** zebrafish, prey capture, calcium imaging, GCaMP, visual perception

## Abstract

Prey capture in zebrafish larvae is an innate behavior which can be observed as early as 4~days postfertilization, the day when they start to swim. This simple behavior apparently involves several neural processes including visual perception, recognition, decision-making, and motor control, and, therefore, serves as a good model system to study cognitive functions underlying natural behaviors in vertebrates. Recent progresses in imaging techniques provided us with a unique opportunity to image neuronal activity in the brain of an intact fish in real-time while the fish perceives a natural prey, paramecium. By expanding this approach, it would be possible to image entire brain areas at a single-cell resolution in real-time during prey capture, and identify neuronal circuits important for cognitive functions. Further, activation or inhibition of those neuronal circuits with recently developed optogenetic tools or neurotoxins should shed light on their roles. Thus, we will be able to explore the prey capture in zebrafish larvae more thoroughly at cellular levels, which should establish a basis of understanding of the cognitive function in vertebrates.

## WHAT CAN WE LEARN FROM THE PREY-CAPTURE BEHAVIOR?

Animal behavior should be adaptive to ever-changing environments, which is essential for survival. This behavioral flexibility is achieved by the cognitive faculty of the brain. In order to study the neural mechanisms underlying cognition and behavior, it is desirable to analyze the activity of individual neurons throughout the brain. Even though the ultimate goal is to understand the human brain, because of the overwhelming number of the neurons (10^12^ neurons) and their connections, reductionist approaches with animal models should be employed to investigate principles of neural functions. A zebrafish larva has approximately 78,000 neurons in a small, transparent brain (Hill et al., 2003), which allows us to observe a wide area of the brain in a single microscopic field and to visualize and manipulate neuronal activity during a behavioral task.

**FIGURE 1:**
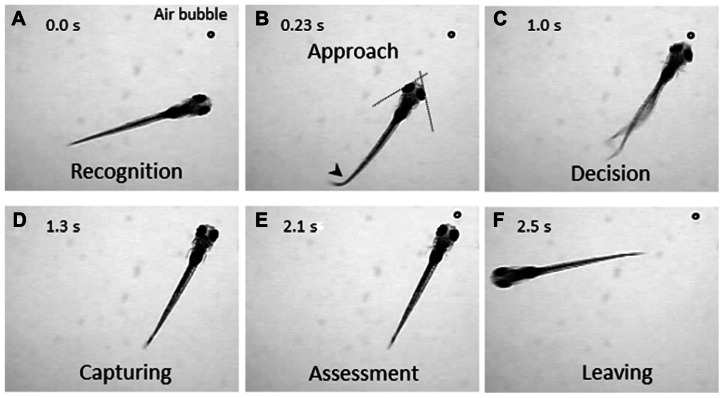
**Prey-capture behavior can be divided into multiple steps of actions.** A 7-day postfertilization (dpf) larva reacts to an air bubble. A possible cognitive or motor process is assigned to each action. **(A)** *Recognition*: An object (an air bubble) comes into sight of a larva and recognized. **(B)** *Approach*: The larva orients its body toward the object with eye convergence (indicated by two crossed dotted lines). An arrowhead indicates J-turn. **(C)** *Decision-making*: The larva makes the final decision to catch the object or abort the behavior. **(D)** *Capturing*: The larva successfully captures the object and put it into the mouth. **(E)** *Assessment*: The larva spits the air bubble out because it is not food. **(F)** *Leaving*: The larva leaves the air bubble to explore other areas.

The zebrafish is a diurnal animal equipped with a highly developed visual system (Branchek, 1984; Branchek and Bremiller, 1984; Easter and Nicola, 1996). Four days after fertilization, zebrafish larvae start swimming and feeding, and capture any potential food. We found that a zebrafish larva shows stereotyped processes of the prey-capture behavior against a small air bubble (**Figure 1**). When a larva perceives the air bubble (**Figure 1A**), the larva initiates the prey-capture behavior; namely, orients itself and exhibits eye convergence (**Figure 1B**). During this orienting behavior, the larva often performs J-turn, bending a far caudal part of the tail to one-side, to fine-tune its position and angle (**Figure 1B**; McElligott and O’Malley, 2005). Then the larva approaches the air bubble as keeping their eyes converged (**Figure 1C**; Bianco et al., 2011), and it captures it (**Figure 1D**). After a successful capture, the larva assesses if it is food or not. If it was not food, the larva spits it out (**Figure 1E**) and swims away from it (**Figure 1F**). In this behavior, there seem to be a couple of decision-making steps: the first step is whether to change its orientation toward the air bubble or to ignore it (**Figures 1A,B**). The final decision is whether to perform the action of catching or to abort the sequence of behaviors (**Figures 1C,D**). The transition from one step to the next step looks probabilistic.

What factors are essential for the larvae to recognize the potential food and making the decision to initiate the prey-capture behavior? How are these decision-making processes modulated by internal states such as hunger or past experience? Through answering these questions, we will be able to get more insights into cognitive functions in the vertebrate brain.

## GCaMP: A SENSITIVE PROBE FOR CALCIUM IMAGING

**FIGURE 2:**
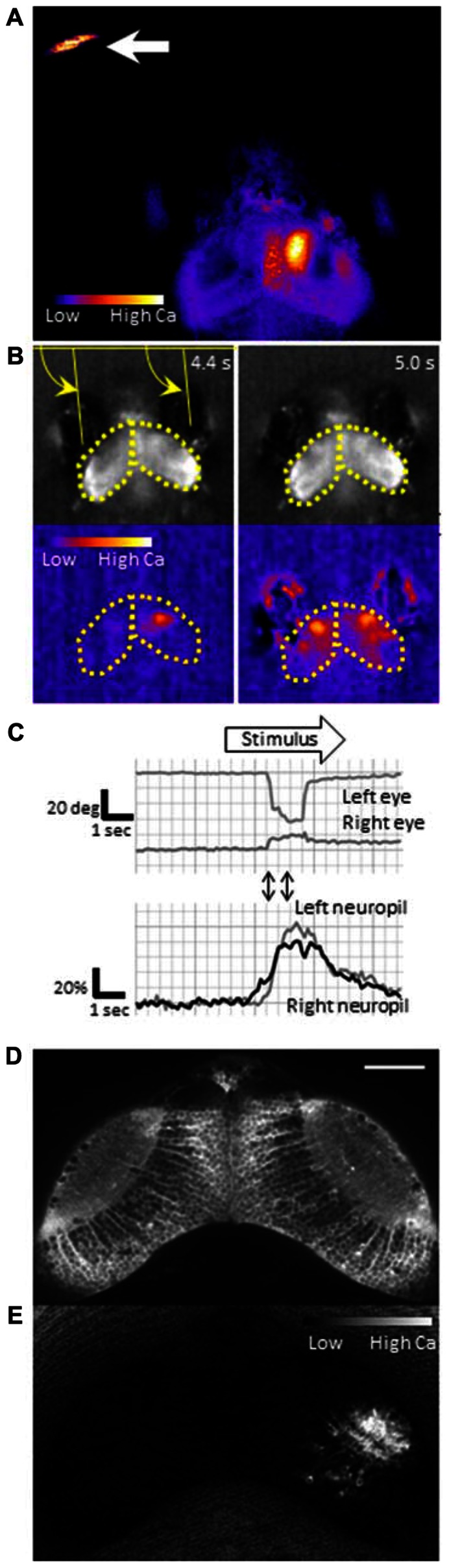
**Functional imaging of the zebrafish larval brain.** GCaMP7a is expressed in the optic tectum of a 7-dpf zebrafish larva embedded in agarose. **(A)** Tectal responses during perception of a swimming paramecium (arrow). Calcium signals are observed in both cell bodies and the neuropil on the right tectum when a paramecium is in the left hemifield. Ratio image was created ad pseudo-colored to show fluorescence changes. **(B)** Tectal responses during perception of a moving spot. A small LCD screen is placed in front of the larva and a moving spot is presented (from left to the right). *Top*: Raw images to show eye positions. *Bottom*: Pseudo-colored images to show fluorescence changes. Yellow arrows indicate eye positions (angles). Yellow dotted lines delineate the optic tectum. **(C)** Eye positions (*top*) and GCaMP7a fluorescence intensity changes (*bottom*) during experiment **(B)**. A moving spot was presented during the time shown as an open arrow. Two-way arrows indicate the time points of 4.4 and 5.0 s shown in **(B)**. The eye convergence (inward movement of both left and right eyes) was evoked by the moving spot. **(D)** Neuronal activity in the optic tectum of a 4-dpf zebrafish larva. A spinning disk confocal microscope (CSU-W1, Yokogawa Electric Corporation, Tokyo, Japan) was used for recording. Scale bar: 50 μm. **(E)** The ratiometric image of **(D)** to reveal the fluorescence change. Small populations of neurons in the right tectum are temporally activated.

To identify neurons that are responsible for the cognitive tasks in the brain, we need a sensitive probe that can report activity in individual neurons *in vivo*. Calcium-sensitive fluorescence probes can measure calcium influx which occurs upon voltage changes in the neurons. Genetically encoded calcium indicators (GECIs) are particularly useful because they can be introduced into neurons of interest using a proper promoter that drives specific expression. GCaMP is a GECI, that consists of circularly permutated enhanced green fluorescent protein (EGFP), calmodulin, and calmodulin-binding peptide M13, and has been widely used for imaging (Nakai et al., 2001). Previously, we generated transgenic zebrafish expressing GCaMP-HS, a modified version of the original GCaMP, and visualized activity of spinal motoneurons during a coiling behavior of an embryo (Muto et al., 2011). However, GCaMP-HS was not sensitive enough to report signals from individual neurons in the optic tectum (Muto, unpublished observation). Therefore, we generated a more sensitive GCaMP, GCaMP7a. Fluorescence changes detected with GCaMP7a during spontaneous neuronal activity in the tectal neuropil, were approximately threefold greater than those detected with GCaMP-HS. By using GCaMP7a, we could visualize neuronal activity in the tectum while the zebrafish larva perceived a paramecium (**Figure 2A**; Muto et al., 2013).

The efforts to improve the signal amplitude and the calcium sensitivity of GCaMP are currently ongoing in several laboratories (Akerboom et al., 2012; Ohkura et al., 2012b; Hoi et al., 2013). The sensitivity of the latest GCaMPs can detect single action potentials *in vivo*. Yet, they may still be less sensitive in comparison to the chemical probe, Oregon Green BAPTA-1 (OGB-1; Akerboom et al., 2012).

## HOW TO IMAGE? FREE-SWIMMING LARVA vs.IMMOBILIZED LARVA

During prey capture against an air bubble or a paramecium, the zebrafish larva exhibits a sequence of discrete motor patterns. So, how can we image the moving brain? It is, in general, very difficult to detect fluorescence intensity changes of moving objects. In the previous study, we successfully detected neuronal activity in a free-swimming larva, mapped locations of the calcium signal in the brain and of the paramecia at the same time, and revealed that activation of the anterior optic tectum is likely to evoke prey-capture behavior (Muto et al., 2013). In this case, we imaged neuronal activity between bouts of swimming activity, that is, when the larva did not move (Muto et al., 2013). It is difficult to image the brain activity of zebrafish larvae in motion. With currently available GCaMPs, the duration of the exposure time required for image acquisition is typically in the order of tens to hundreds of milliseconds. This exposure time gives only blurred image when the object is moving. To image a moving larva, much brighter fluorescence probes and more sensitive cameras are required.

An alternative approach to image the brain activity is the use of a partially restrained larva; namely, its head and trunk are fixed in agarose while its eyes and tail are free. In this condition, two defining features of prey capture, eye convergence and J-turns, can be observed (Bianco et al., 2011). The merit of this setup is that, one can present any visual stimuli on a liquid-crystal display (LCD) screen that may mimic an air bubble or a paramecium. Because motionless objects are not perceived in the visual system of a larva (Muto et al., 2013), the stimulus to be presented should contain a motion component, which maybe direct (e.g., a moving spot which mimics a paramecium) or relative (e.g., a stationary spot on a moving background, which mimics an air bubble). As shown in **Figure 2**, a moving spot could evoke both neuronal activity in the tectum and the eye convergence in the partially restrained larva. The eye convergence is an initial step of the sequential behaviors (**Figure 1B**). The succeeding steps of prey capture can also be investigated in a closed-loop virtual reality setup (Trivedi and Bollmann, 2013). Thus, we can study multiple steps of prey capture in a partially restrained larva. What visual cues are more likely to evoke prey recognition? Which neurons are activated during the prey recognition? How will these neuronal activities be changed before and after the larva learned that the air bubble was not food?

Pioneering ethological study by Ewert (1980) showed that, in prey catching behavior, toads preferred visual stimuli that resembled a shape and moving pattern of a worm, in contrast to the same shape rotated by 90°. In zebrafish, preference for size and speed of a moving spot in prey-capture behavior has been reported (Bianco et al., 2011). In medaka fish, a moving object with pink noise component was more potent to evoke feeding behavior (Matsunaga and Watanabe, 2012). These findings should be taken account into the parameters to create a “virtual paramecia” on the LCD screen.

## TOWARD ELUCIDATION OF THE ENTIRE FUNCTIONAL NEURAL CIRCUITS FOR PREY CAPTURE

The optic tectum has a laminar structure; superficial layers that receive sensory input and deeper layers that are involved in motor output (Salas et al., 1997). Visual information processing for prey recognition starts at the most superficial layer, the stratum opticum (SO) in the tectum. Del Bene et al. (2010) discovered that a subclass of GABAergic interneurons located in the SO responded preferentially to visual stimuli with larger spatial frequency, and was indispensable for recognition of small objects and paramecia. We predicted functional connections between the anterior tectum area and the motor pathway that generates approach swimming (Muto et al., 2013). Identification of the neural pathway(s) that follows the initial stages of visual perception is the target of the future study. Gahtan et al. (2005) found that a pair of reticulospinal neurons, namely, MeLc (caudal medial–lateral) and MeLr (rostral medial–lateral) in the nucleus of the medial longitudinal fasciculus of the midbrain tegmentum is essential for prey capture, specifically orienting behavior. These reticulospinal neurons extend their dendrites toward the ventral tectum, which suggests that they convey the output from the tectum to the motor system (Gahtan et al., 2005). It is unclear whether these identified pathways play a role in prey recognition itself (e.g., pattern recognition of food) or in up- or downstream of it (e.g., specifying the range of possible food size, or relaying the motor command for prey capture). The neural pathway(s) that follows the initial stages of visual perception will be identified by examining neuronal activity of the entire brain using pan-neuronal GCaMP expression (Ahrens et al., 2012) and also investigating specific populations of neurons in which the GCaMP is expressed via Gal4-UAS system (Kawakami et al., 2010).

## TECHNICAL REQUIREMENTS FOR THE FUTURE STUDY

To explore neuronal activity in wide areas of the brain by calcium imaging, it is necessary to achieve single-cell resolution deep in the brain with high acquisition rates. Fulfilling this requirement is still a technical challenge. Fluorescent compound microscopy can generate real-time images (typically, ~10 fps in our recording set up), but only detect calcium signals near the surface of the tectum from a dorsal side (**Figures 2A,B**). It does not give enough resolution along the *z*-axis to separate signals from overlapping neurons. Two-photon scanning microscopy gives much deeper light penetration with excellent image quality. The drawback is a slow frame acquisition rate due to the slow laser scanning (a few frames per second; Ahrens et al., 2012). A spinning-disk confocal microscope can achieve a higher temporal resolution, and onset of the calcium rise could be determined with a precision of up to several milliseconds (Takahashi et al., 2007). We could detect neuronal activity at a single-cell resolution in the optic tectum (**Figures 2D,E**). The use of light sheet microscopy may solve both penetration and temporal resolution problems, and has been applied to the entire brain imaging (Huisken, 2012; Ahrens et al., 2013). Another critical issue in calcium imaging of the visual system is how to minimize undesirable retinal stimulation by the excitation light. *nacre* mutants have been commonly used because the lack of melanophores in this mutant allows light penetration which is necessary for brain imaging, whereas the intact retinal pigment epithelia block the scattered excitation light coming from the back of the retina (Sumbre et al., 2008; Muto et al., 2013). The amount of excitation light should be minimized so that it does not interfere with the visual stimulus.

Once we identify neuronal circuits activated during a prey-capture behavior, we need to manipulate their activity to prove necessity and sufficiency. Necessity can be tested by blocking neuronal activity with a neurotoxin (Asakawa et al., 2008) or optogenetic tools such as halorhodopsin (Arrenberg et al., 2010). Sufficiency can be tested by optical activation of the identified neurons with light-gated glutamate receptors or channelrhodopsin-2 (Douglass et al., 2008; Wyart et al., 2009; Arrenberg et al., 2010). These effectors can be genetically expressed using a tissue-specific promoter (Faraco et al., 2006), the regulatory elements located on a bacterial artificial chromosome (BAC; Suster et al., 2011), or Gal4 driver lines in combination with UAS constructs (Asakawa et al., 2008). A possible limitation is that expression of effector genes may not be restricted to specific neuronal circuits, rather, they are often expressed in multiple regions. To achieve specific expression of effectors that inhibit neuronal activity, it will be necessary to combine two expression systems, e.g., Cre/loxP and Gal4-UAS (Sato et al., 2007). Specificity in the optogenetic activation can also be achieved by spatially restricted illuminations. Fiber optics can be used to illuminate a small area by choosing an appropriate fiber size (Arrenberg et al., 2009). Spatially patterned illumination can be achieved with a digital micromirror device (DMD; Zhu et al., 2012) or by digital holography (Oron et al., 2012).

The effect of neuronal activation by channelrhodopsin-2 or inhibition by halorhodopsin should be confirmed by calcium imaging, which requires the simultaneous use of an optogenetic tool and a calcium probe. Both GCaMP and channelrhodopsin-2 require blue light, and therefore cannot be used at the same time. Color-shifted GCaMPs have been developed (Zhao et al., 2011; Ohkura et al., 2012a; Walker et al., 2013), and their usefulness *in vivo* in transgenic animals should be investigated and demonstrated.

In conclusion, prey capture in a zebrafish larva involves cognitive processes and, therefore, serves as an excellent model to visualize higher brain functions at a cellular level. As a first step, we visualized neuronal activity at the initial step of the prey-capture behavior, prey perception (Muto et al., 2013). Further imaging studies will reveal the entire functional neuronal circuits that are activated during this behavior. In combination with recent technology advances including optogenetic tools, we will obtain more insights into basic principles of computational and cognitive properties of the brain.

## Conflict of Interest Statement

The authors declare that the research was conducted in the absence of any commercial or financial relationships that could be construed as a potential conflict of interest.

## References

[B1] Ahrens M. B. Li J. M. Orger M. B. Robson D. N. Schier A. F. Engert F.(2012) Brain-wide neuronal dynamics during motor adaptation in zebrafish. *Nature* 485 471–477 10.1038/nature11057 22622571 PMC3618960

[B2] Ahrens M. B. Orger M. B. Robson D. N. Li J. M. Keller P. J. (2013) Whole-brain functional imaging at cellular resolution using light-sheet microscopy. *Nat. Methods* 10 413–420 10.1038/nmeth.2434 23524393

[B3] Akerboom J. Chen T. W. Wardill T. J. Tian L. Marvin J. S. Mutlu S.(2012) Optimization of a GCaMP calcium indicator for neural activity imaging. *J. Neurosci.* 32 13819–13840 10.1523/JNEUROSCI.2601-12.2012 23035093 PMC3482105

[B4] Arrenberg A. B. Del Bene F. Baier H. (2009) Optical control of zebrafish behavior with halorhodopsin. *Proc. Natl. Acad. Sci. U.S.A.* 106 17968–17973 10.1073/pnas.0906252106 19805086 PMC2764931

[B5] Arrenberg A. B. Stainier D. Y. Baier H. Huisken J. (2010) Optogenetic control of cardiac function. *Science* 330 971–974 10.1126/science.1195929 21071670

[B6] Asakawa K. Suster M. L. Mizusawa K. Nagayoshi S. Kotani T. Urasaki A.(2008) Genetic dissection of neural circuits by Tol2 transposon-mediated Gal4 gene and enhancer trapping in zebrafish. *Proc. Natl. Acad. Sci. U.S.A.* 105 1255–1260 10.1073/pnas.0704963105 18202183 PMC2234125

[B7] Bianco I. H. Kampff A. R. Engert F. (2011) Prey capture behavior evoked by simple visual stimuli in larval zebrafish. *Front Syst. Neurosci. * 5: 101 10.3389/fnsys.2011.00101 PMC3240898 22203793

[B8] Branchek T. (1984) The development of photoreceptors in the zebrafish, *Brachydanio rerio*. II. Function. *J. Comp. Neurol.* 224 116–122 10.1002/cne.902240110 6715575

[B9] Branchek T. Bremiller R. (1984) The development of photoreceptors in the zebrafish, *Brachydanio rerio*. I. Structure. *J. Comp. Neurol.* 224 107–115 10.1002/cne.902240109 6715574

[B10] Del Bene F. Wyart C. Robles E. Tran A. Looger L. Scott E. K.>(2010) Filtering of visual information in the tectum by an identified neural circuit. *Science* 330 669–673 10.1126/science.1192949 21030657 PMC3243732

[B11] Douglass A. D. Kraves S. Deisseroth K. Schier A. F. Engert F. (2008) Escape behavior elicited by single, channelrhodopsin-2-evoked spikes in zebrafish somatosensory neurons. *Curr. Biol.* 18 1133–1137 10.1016/j.cub.2008.06.077 18682213 PMC2891506

[B12] Easter S. S. Jr. Nicola G. N. (1996) The development of vision in the zebrafish (*Danio rerio*). *Dev. Biol.* 180 646–663 10.1006/dbio.1996.0335 8954734

[B13] Ewert J. P. (1980) *Neuroethology*. Berlin: Springer-Verlag. 10.1007/978-3-642-67500-3

[B14] Faraco J. H. Appelbaum L. Marin W. Gaus S. E. Mourrain P. Mignot E. (2006) Regulation of hypocretin (orexin) expression in embryonic zebrafish. *J. Biol. Chem.* 281 29753–29761 10.1074/jbc.M605811200 16867991

[B15] Gahtan E. Tanger P. Baier H. (2005) Visual prey capture in larval zebrafish is controlled by identified reticulospinal neurons downstream of the tectum. *J. Neurosci.* 25 9294–9303 10.1523/JNEUROSCI.2678-05.2005 16207889 PMC6725764

[B16] Hill A. Howard C. V. Strahle U. Cossins A. (2003) Neurodevelopmental defects in zebrafish (*Danio rerio*) at environmentally relevant dioxin (TCDD) concentrations. *Toxicol. Sci.* 76 392–399 10.1093/toxsci/kfg241 14600291

[B17] Hoi H. Matsuda T. Nagai T. Campbell R. E. (2013) Highlightable Ca(2+) indicators for live cell imaging. *J. Am. Chem. Soc.* 135 46–49 10.1021/ja310184a 23256581

[B18] Huisken J. (2012) Slicing embryos gently with laser light sheets. *Bioessays* 34 406–411 10.1002/bies.201100120 22396246

[B19] Kawakami K. Abe G. Asada T. Asakawa K. Fukuda R. Ito A.(2010) zTrap: zebrafish gene trap and enhancer trap database. *BMC Dev. Biol.* 10: 105. 10.1186/1471-213X-10-105 PMC2970601 20950494

[B20] Matsunaga W. Watanabe E. (2012) Visual motion with pink noise induces predation behaviour. *Sci. Rep.* 2 219 10.1038/srep00219 PMC3255084 22355733

[B21] McElligott M. B O’Malley D. M. (2005) Prey tracking by larval zebrafish: axial kinematics and visual control. *Brain Behav. Evol.* 66 177–196 10.1159/000087158 16088102

[B22] Muto A. Ohkura M. Abe G. Nakai J. Kawakami K. (2013) Real-time visualization of neuronal activity during perception. *Curr. Biol.* 23 307–311 10.1016/j.cub.2012.12.040 23375894

[B23] Muto A. Ohkura M. Kotani T. Higashijima S. Nakai J. Kawakami K. (2011) Genetic visualization with an improved GCaMP calcium indicator reveals spatiotemporal activation of the spinal motor neurons in zebrafish. *Proc. Natl. Acad. Sci. U.S.A.* 108 5425–5430 10.1073/pnas.1000887108 21383146 PMC3069178

[B24] Nakai J. Ohkura M. Imoto K. (2001) A high signal-to-noise Ca(2+) probe composed of a single green fluorescent protein. *Nat. Biotechnol.* 19 137–141 10.1038/84397 11175727

[B25] Ohkura M. Sasaki T. Kobayashi C. Ikegaya Y. Nakai J. (2012a) An improved genetically encoded red fluorescent Ca^2^^+^ indicator for detecting optically evoked action potentials. *PLoS ONE * 7: e39933. 10.1371/journal.pone.0039933 PMC3393713 22808076

[B26] Ohkura M. Sasaki T. Sadakari J. Gengyo-Ando K. Kagawa-Nagamura Y. Kobayashi C. (2012b) Genetically encoded green fluorescent Ca^2^^+^ indicators with improved detectability for neuronal Ca^2^^+^ signals. *PLoS ONE * 7:e51286 10.1371/journal.pone.0051286 PMC3519846 23240011

[B27] Oron D. Papagiakoumou E. Anselmi F. Emiliani V. (2012) Two-photon optogenetics. *Prog. Brain Res.* 196 119–143 10.1016/B978-0-444-59426-6.00007-0 22341324

[B28] Salas C. Herrero L. Rodriguez F. Torres B. (1997) Tectal codification of eye movements in goldfish studied by electrical microstimulation. f. *Neuroscience* 78 271–288 10.1016/S0306-4522(97)83048-5 9135107

[B29] Sato T. Hamaoka T. Aizawa H. Hosoya T. Okamoto H. (2007) Genetic single-cell mosaic analysis implicates ephrinB2 reverse signaling in projections from the posterior tectum to the hindbrain in zebrafish. *J. Neurosci.* 27 5271–5279 10.1523/JNEUROSCI.0883-07.2007 17507550 PMC6672335

[B30] Sumbre G. Muto A. Baier H. Poo M. M. (2008) Entrained rhythmic activities of neuronal ensembles as perceptual memory of time interval. *Nature* 456 102–106 10.1038/nature07351 18923391 PMC2896960

[B31] Suster M. L. Abe G. Schouw A. Kawakami K. (2011) Transposon-mediated BAC transgenesis in zebrafish. *Nat. Protoc.* 6 1998–2021 10.1038/nprot.2011.416 22134125

[B32] Takahashi N. Sasaki T. Usami A. Matsuki N. Ikegaya Y. (2007) Watching neuronal circuit dynamics through functional multineuron calcium imaging (fMCI). *Neurosci. Res.* 58 219–225 10.1016/j.neures.2007.03.001 17418439

[B33] Trivedi C. A. Bollmann J. H. (2013) Visually driven chaining of elementary swim patterns into a goal-directed motor sequence: a virtual reality study of zebrafish prey capture. *Front. Neural Circuits* 7:86 10.3389/fncir.2013.00086 PMC3650304 23675322

[B34] Walker A. S. Burrone J. Meyer M. P. (2013) Functional imaging in the zebrafish retinotectal system using RGECO. *Front Neural Circuits * 7:34 10.3389/fncir.2013.00034 PMC3589694 23508811

[B35] Wyart C. Del Bene F. Warp E. Scott E. K. Trauner D. Baier H.>(2009) Optogenetic dissection of a behavioural module in the vertebrate spinal cord. *Nature* 461 407–410 10.1038/nature08323 19759620 PMC2770190

[B36] Zhao Y. Araki S. Wu J. Teramoto T. Chang Y. F. Nakano M.>(2011) An expanded palette of genetically encoded Ca(2)(+) indicators. *Science* 333 1888–1891 10.1126/science.1208592 21903779 PMC3560286

[B37] Zhu P. Fajardo O. Shum J. Zhang Scharer Y. P. Friedrich R. W. (2012) High-resolution optical control of spatiotemporal neuronal activity patterns in zebrafish using a digital micromirror device. *Nat. Protoc.* 7 1410–1425 10.1038/nprot.2012.072 22743832

